# Hedonic Deficits in Parkinson’s Disease: Is Consummatory Anhedonia Specific?

**DOI:** 10.3389/fneur.2014.00024

**Published:** 2014-03-10

**Authors:** Gwenolé Loas, Cécile Duru, Olivier Godefroy, Pierre Krystkowiak

**Affiliations:** ^1^Department of Psychiatry, Amiens University Hospital, Amiens, France; ^2^EA 4559, Laboratoire de Neurosciences Fonctionnelles et Pathologie, University of Picardie Jules Verne, Amiens, France; ^3^Department of Neurology, Amiens University Hospital, Amiens, France

**Keywords:** anhedonia, depression, dopamine, Parkinson’s disease

## Abstract

**Background:** Anhedonia, the lowered ability to experience pleasure, is one of the non-motor symptoms of Parkinson’s disease (PD) that is underdiagnosed and consequently undertreated. Few studies have investigated anhedonia in PD by taking into account the influence of socio-demographic variables and versus a control group composed of patients with a pure motor neurologic disease other than PD. The aim of this study was to investigate hedonic deficits in patients with PD compared to a control group of patients with non-Parkinson motor neurologic disease (OND), matched for age, gender, level of education, and inpatient/outpatient status. Distinctions between anticipatory and consummatory anhedonia and between endogenomorphic and non-endogenomorphic depression were taken into account.

**Methods:** The study population comprised 49 PD patients and 40 subjects with OND. Anhedonia was rated by using the anticipatory [Temporal Experience Pleasure Scale (TEPS)-ANT] and consummatory (TEPS-CONS) subscales of the TEPS and two subscales extracted from the revised Physical Anhedonia Scale (PAS), measuring physical anticipatory (PAS-ANT) and physical consummatory (PAS-CONS) anhedonias. The Snaith–Hamilton Pleasure Scale (SHAPS) and the Beck Depression Inventory-II (BDI-II) were also used together with a subscale extracted from the BDI-II (ENDO-BDI-II) for the diagnosis of endogenomorphic depression. Statistical analyses were performed on the whole group and on the PD group.

**Results:** As hypothesized, several anhedonia scores varied with age and gender in the whole population or in the PD group. On univariate or multivariate analyses, only PAS-CONS was specific for PD and only SHAPS scores differed between depression subtypes in the whole population or the PD group.

**Conclusion:** This study suggests that physical consummatory anhedonia could be specific to PD subjects.

## Introduction

Anhedonia is defined as a lowered ability to experience pleasure. It is recognized to be a core symptom of major depression and approximately 30–40% of subjects with Parkinson’s disease (PD) experience significant depression (1). However, independently of the presence of depression in PD, anhedonia can be considered to be a specific mood disorder explained by hypoactivation of the dopamine reward pathway secondary to the degenerative processes observed in PD, particularly in the mesolimbic area (2). In PD, anhedonia is considered as one of the hypodopaminergic symptoms, some treatments for PD, especially dopamine agonists, can induce impulse control disorders (ICD) such as pathologic gambling or hypersexuality, that, in contrast, are characterized by hyperhedonia.

Anhedonia, as well as other non-motor symptoms of PD, is underdiagnosed and consequently undertreated (3). It is therefore surprising that although investigation of anhedonia in PD could have important clinical and therapeutic consequences, few studies have been published on this subject.

One of the main unresolved questions is whether or not the relationship between anhedonia and PD can be completely explained by the relationship between depression and anhedonia. This relationship has been extensively examined in the psychiatric literature. Anhedonia is present in more than two-thirds of all individuals with major depression (4). Non-major depression is associated with a low prevalence of anhedonia and the hedonic capacity may remain intact.

The prevalence of anhedonia in PD ranged from 4.7 to 45.7% using a cutoff of 3 on the Snaith–Hamilton Pleasure Scale (SHAPS) (5–12).

A strong association in PD was observed between depression and anhedonia, but the studies reported discordant results concerning the level of anhedonia in non-depressed PD patients, as anhedonia appears to be associated with akinesia, apathy, and cognitive impairment, but these relationships were not reported in all studies (5–12).

In major depression, several studies have validated the hypothesis proposed by Klein (13) suggesting the existence of a subtype of major depression called endogenomorphic depression characterized by pervasive anhedonia. According to Klein, endogenomorphic depression is characterized by consummatory anhedonia and appetitive (anticipatory) anhedonia, whereas non-endogenomorphic depression is characterized exclusively by appetitive anhedonia. Recently, the distinction between the experiences of pleasure related either to online experience in response to a specific stimulus (consummatory pleasure) or to future pleasurable activities (appetitive or anticipatory pleasure) has been validated by cumulative, cross-discipline evidence (14). According to Burgdorf and Panksepp (15), there is now converging evidence to suggest that various regions of the limbic system, especially ventral striatal dopaminergic systems, are involved in an anticipatory (appetitive) positive affective state. Dopaminergic-independent mechanisms, mediated by opiate and GABA receptors in the ventral striatum, amygdala, and orbital frontal cortex, play an important role in elaborating consummatory positive affective (i.e., sensory pleasure) states, and various neuropeptides mediate homeostatic satisfactions.

Several hypotheses were tested in the present study. Firstly, is one of the various types of anhedonia specific to PD? Secondly, we tested the hypothesis that, in PD, anticipatory and consummatory anhedonias were associated with non-endogenomorphic and endogenomorphic depression, respectively. Thirdly, is there a specific pattern of association in PD between features of the disease and the various types of anhedonia that explain the discrepancies reported in the literature?

The aim of this study was therefore to investigate hedonic deficits in PD by taking into account the limitations of previous studies. The three previous hypotheses were tested. We used several rating scales evaluating the various types of anhedonia, notably the distinction between consummatory and anticipatory anhedonia. A control group comprising subjects with various non-PD motor neurologic diseases with control of socio-demographic variables was used. The relationships between the various types of anhedonia and depression were examined by taking into account the non-endogenomorphic/endogenomorphic distinction.

## Materials and Methods

### Patients

The study included 49 patients with idiopathic PD according to the UKPDSBB (United Kingdom Parkinson’s Disease Brain Bank) criteria and 40 patients with a non-PD motor neurologic disease (OND). Participants were consecutively recruited in the Amiens University Hospital Department of Neurology. All subjects were outpatients and the study was performed at follow-up visits during regular treatment with antiparkinsonian drugs. The study was approved by our local Ethics Committee and, in accordance with the Helsinki declaration, each subject signed an informed consent form before starting the study.

All patients with idiopathic PD or OND were over the age of 18 and were not demented (Mini-mental State Examination, MMSE >23). The diagnosis of idiopathic PD was established by a neurologist specialized in movement disorders (Pierre Krystkowiak). Exclusion criteria were: history of non-neurological chronic disease, history of psychiatric disorder, or neurologic disease other than idiopathic PD, non-stabilized dopamine therapy. The following data were available for PD patients: MMSE, motor examination (UPDRS part III), Hoehn and Yahr stage, age at onset, duration of disease, fluctuations (yes or no), and treatment (l-DOPA, dopamine agonists, entacapone).

The control group comprised 40 patients with the following motor neurologic diseases: cervical dystonia (*N* = 17), hemifacial spasm (*N* = 8), blepharospasm (*N* = 7), writer’s cramp (*N* = 4), cerebellar ataxia (*N* = 2), myoclonus (*N* = 1), and congenital torticollis (*N* = 1). As dystonia is considered as basal ganglia disorder contrary to the diagnoses of the other controls, these two groups were compared on the socio-demographic, clinical, and psychometrical variables. No significant differences were found.

### Rating scales

The subjects of the two groups filled out four questionnaires, three rating anhedonia and one rating depression.

Consummatory and anticipatory anhedonia were rated using the Temporal Experience Pleasure Scale (TEPS) and two subscales extracted from the revised Physical Anhedonia Scale (PAS).

– TEPS: The 18-item Temporal Experience Pleasure Scale (TEPS) (14) comprises two subscales measuring trait consummatory pleasure (consummatory subscale of the Temporal Experience of Pleasure Scale, TEPS-CONS) and trait pleasure in anticipation of future events (anticipatory subscale of the TEPS, TEPS-ANT). The TEPS-CONS and TEPS-ANT comprise 16 and 10 items, respectively, rated from 1 to 6 with total scores ranging from 16 to 96 and 10 to 60 for the TEPS-CONS and TEPS-ANT, respectively. The level of anhedonia is inversely related to the total score of the rating scales. The psychometric properties of the TEPS have been found to be satisfactory in various samples of non-clinical and psychiatric patients (14, 16). French versions of these scales, with satisfactory psychometric properties, were used in this study (17).
– Subscales of the revised PAS (18): several studies (14, 16, 17) in non-clinical and clinical subjects have reported that the TEPS-ANT and TEPS-CONS were significantly correlated with the PAS, whereas the revised Social Anhedonia Scale (SAS) was only correlated with the TEPS-ANT. Using the university sample of the French version of the TEPS validation study (17), we therefore created the PAS anticipatory (PAS-ANT) and PAS consummatory (PAS-CONS) scales by taking into account the correlations between the items of the PAS and the TEPS-CONS or TEPS-ANT scales. Ten items and 16 items were adopted for the PAS-ANT and PAS-CONS subscales, respectively. The items of the PAS-ANT and PAS-CONS were: PAS-ANT (5, 9, 18, 19, 23, 25, 48, 56, 59, 61); PAS-CONS (4, 11, 13, 21, 22, 24, 29, 32, 35, 46, 50, 52, 55, 57, 8, 60) using the pages 642–643 of the publication of the French version of the PAS (19). These two subscales presented satisfactory psychometric properties (17). Total scores for the PAS-ANT and PAS-CONS ranged from 0 to 10 and 0 to 16, respectively, and were related to the level of anhedonia. PAS-ANT and PAS-CONS rate the anticipatory and consummatory components of physical anhedonia, respectively.
– SHAPS: The SHAPS (20) is the only rating scale meeting the criteria of a “suggested” anhedonia scale according to the Movement Disorder Society (21). The SHAPS is a self-rating scale comprising 14 items that cover four domains of pleasure: interest/pastimes; social interaction; sensory experiences, and food/drink. High scores represent a reduction of hedonic tone. A score of three or more allows a categorical definition of anhedonia (20). The French version of the scale with satisfactory reliability and validity was used (22). In view of the correlations between the SHAPS and the TEPS-ANT and TEPS-CONS reported in one study (17), the SHAPS was considered to be a measure of consummatory anhedonia and anticipatory anhedonia.
– Contrary to the TEPS, the PAS-ANT and PAS-CONS allowed to measure the physical and sensorial components of anticipatory and consummatory anhedonias. The use of the SHAPS allowed firstly to study the prevalence of anhedonia as cutoff scores were available and secondly to compare the results of the present study with these reported in the previous studies.
– BDI-II: Depression was rated using the 21-item Beck Depression Inventory-II [BDI-II (23). The BDI-II is the 1996 revision of the classic BDI. The French version of the BDI-II has satisfactory psychometric properties (24). The total score ranges from 0 to 63 and higher total scores indicate more severe depressive symptoms. The following cutoff scores were used to distinguish non-depressed, moderately depressed, and severely depressed subjects: 0–11: no depression; 12–28: mild and moderate depression; and 29–63: severe depression. A subscale (endogenomorphic BDI-II subscale, ENDO-BDI-II) comprising five items (loss of satisfaction, loss of interest in people, loss of interest in sex, early morning wakening, and loss of appetite) has also been constructed (25) to allow the diagnoses of endogenous (endogenomorphic) depression. The total score of this subscale ranges from 0 to 15 and the following cutoff scores have been defined: no depression (0–3), non-endogenomorphic depression (4–6), endogenomorphic depression (7–15).
– The following rating scales or interviews were used to evaluate the clinical features of PD patients: MMSE with scores ranging from 0 to 30 (worst score = 0; best score = 30); motor score from the UPDRS (motor part of the Unified PD Rating Scale; worst score = 108, best score = 0). Hoehn and Yahr stage was used to rate severity of the disease (worst score = 5; best score = 0).

### Statistical analysis

*Firstly*, to test the hypothesis that one subtype of anhedonia may be specific to PD and, secondly, to test the hypothesis of specific associations between each subtype of anhedonia and depression, we examined the relationships, for the two groups combined, between each anhedonia scale and socio-demographic or clinical variables. Univariate and multivariate analyses were performed using the following variables: group (PD versus OND), gender, age, level of education, depression severity (BDI score), subtypes of depression (non-depressed, mild depression, severe depression), endogenomorphic subtypes of depression (non-depressed, non-endogenomorphic, endogenomorphic depression). For univariate analyses, Student’s *t* tests, analysis of variance (ANOVA), Pearson’s correlation, and Chi-square analysis were used. Multivariate analyses were performed for each anhedonia scale. Multiple regression analysis was conducted using the anhedonia scores as dependent variables and the significant variables identified on univariate analyses as independent variables. A less conservative *p* (*p* < 0.1) was used for inclusion in multivariate analyses.

*Secondly*, the relationships between the various types of anhedonia and depression were examined in each group according to the two different subtypes of depression.

*Thirdly*, the relationships between the various types of anhedonia and features of the disease in the PD group were examined by univariate and multivariate analyses using the following variables: gender, age, level of education, depression severity (BDI score), subtypes of depression (non-depressed, mild depression, severe depression), endogenomorphic subtypes of depression (non-depressed, non-endogenomorphic, endogenomorphic depression), MMSE, motor UPDRS, Hoehn and Yahr stage, age at onset, duration of disease, fluctuations (yes or no), and treatment (l-DOPA, dopaminergic agonists, entacapone). Multiple regressions were conducted using the anhedonia scores as dependent variables and the significant variables identified on univariate analyses as independent variables. A less conservative *p* (*p* < 0.1) was used for inclusion in multivariate analyses. Statistical tests were two-tailed with *p* < 0.05.

## Results

The clinical and psychometric characteristics of the two groups are shown in Table 1.

**Table 1:** **Comparison between Parkinson’s disease (PD) subjects and subjects with other neurologic disease (OND)**.

	PD (*n* = 49)	OND (*n* = 40)	*P*
Gender	57% males (*n* = 28)	45% males (*n* = 18)	0.25
Age (years)	64.84 (SD = 10.84)	60.1 (SD = 12.07)	0.06
Level of education^a^ (I/II)	22/14/13	13/14/13	0.67
TEPS-ANT	39.37 (SD = 6.34)	39.3 (SD = 6.65)	0.96
TEPS-CONS	34.65 (SD = 6.72)	34.95 (SD = 7.44)	0.84
PAS-ANT	2.39 (SD = 1.55)	2.27 (SD = 1.36)	0.72
PAS-CONS	5.67 (SD = 2.19)	4.7 (SD = 2.26)	0.04^1^
SHAPS	1.24 (SD = 1.53)	0.95 (SD = 1.45)	0.36
BDI-II total score	18.51 (SD = 8.9)	15 (SD = 11.59)	0.11
SHAPS ≥3	18.4% (*n* = 9)	7.5% (*n* = 3)	0.12
Depression (BDI-II >11)	75.5% (*n* = 37)	50% (*n* = 20)	0.019^1^
Endogenomorphic depression	14.3% (*n* = 7)	12.5% (*n* = 5)	0.73
(ENDO-BDI-II >6)			
Duration of disease (years)	7.37 (SD = 4.56)	7.2 (SD = 5.3)	0.8
Age at onset (years)	56.7 (SD = 10.44)	55.5 (SD = 9.3)	0.5
MMSE	25.29 (3.27)		
UPDRS part III	29.36 (32.16)		
Hoehn and Yahr stage	2.25 (0.8)		
Fluctuations	67.3% (*N* = 33)		
l-DOPA	87.7% (*N* = 43)		
Dopamine agonists	63.3% (*N* = 31)		
Entacapone	36.7% (*N* = 18)		

*^a^ Level of education: I < baccalaureate, II = baccalaureate; III > baccalaureate; baccalaureate corresponds to the end of secondary school studies, i.e., 12 years of accredited education after the age of 6; TEPS, Temporal Experience of Pleasure Scale; TEPS-ANT, anticipatory subscale of the TEPS; TEPS-CONS, consummatory subscale of the TEPS; PAS, revised Physical Anhedonia Scale; PAS-ANT, anticipatory subscale of the PAS; PAS-CONS, consummatory subscale of the PAS; SHAPS, Snaith–Hamilton Pleasure Scale; BDI-II, Beck Depression Inventory-II; MMSE, Mini-Mental State Examination; fluctuations (Levodopa-related motor complications); ^1^*p* < 0.05*.

### Entire sample (two groups of patients; *N* = 89)

For the entire sample, the univariate associations between each anhedonia scale and socio-demographic variables and depression are shown in Table 2.

**Table 2:** **Univariate associations between socio-demographic variables, depression, and anhedonia scales for the entire sample (*N* = 89) or for the Parkinson’ group (*N* = 49)**.

	TEPS-ANT		TEPS-CONS		PAS-ANT		PAS-CONS		SHAPS	
	89	49	89	49	89	49	89	49	89	49
Gender					0.07	0.09				
Age (years)						0.1	0.1	**0.05**		**0.05**
BDI-II total score			**0.05**						**0.05**	
Group (PD/C)^a^							**0.04**			
Depression subtype^b^									**0.015**	**0.02**
Endogenomorphic subtype^c^									**0.05**	0.1
MMSE				**0.05**						
UPDRS-III		**0.05**								
Hoehn and Yahr stage		**0.05**						**0.05**		
Age of onset						**0.05**		**0.05**		**0.05**
Duration				**0.05**						**0.05**
Entacapone						0.1				

*TEPS, Temporal Experience of Pleasure Scale; TEPS-ANT, anticipatory subscale of the TEPS; TEPS-CONS, consummatory subscale of the TEPS; PAS, revised Physical Anhedonia Scale; PAS-ANT, anticipatory subscale of the PAS; PAS-CONS, consummatory subscale of the PAS; SHAPS, Snaith–Hamilton Pleasure Scale; BDI-II, Beck Depression Inventory-II*.

^a^ Group (PD, Parkinson’s disease group; OND, non-Parkinson’s motor neurologic disease),

^b^ (non-depressed BDI-II: 0–11, mild depressed: BDI-II: 12–28, severely depressed: BDI-II: 29–63);

*^c^ endogenomorphic subtypes using endogenomorphic subscale of the BDI-II (ENDO-BDI-II): no depression (ENDO-BDI-II: 0–3), non-endogenomorphic depression (ENDO-BDI-II: 4–6), endogenomorphic depression (ENDO-BDI-II: 7–15). Bold font denotes *p* < 0.05*.

#### Univariate analyses

Several associations between gender or age and anhedonia scales were observed, the majority of which was not significant. In terms of the specificity for anhedonia, only PAS-CONS scores were significantly different between groups, with higher scores observed in PD patients compared to controls. A trend toward a significant correlation was also observed between age and PAS-CONS (*r* = 0.19, *p* < 0.1).

Concerning the relationship between depression and anhedonia, two anhedonia scales’ (TEPS-CONS and SHAPS) scores were correlated with BDI-II scores, with values of −0.21 (*p* < 0.05) and 0.26 (*p* < 0.05), respectively. When categorical definitions of depression were used, only SHAPS scores were correlated with depression.

Significant differences for SHAPS scores were observed between non-depressed, mildly depressed, and severely depressed subjects [*F*(2, 86) = 4.42, *p* = 0.015]. *Post hoc* tests showed that severely depressed subjects had significantly higher SHAPS scores than mildly depressed subjects and non-depressed subjects. No significant difference was observed between mildly depressed subjects and non-depressed subjects.

Significant differences for SHAPS scores were observed between non-depressed, non-endogenomorphic, and endogenomorphic depressed subjects [*F*(2, 86) = 3.1, *p* = 0.05]. *Post hoc* tests showed that endogenomorphic depressed subjects had significantly higher SHAPS scores than non-endogenomorphic depressed subjects and non-depressed subjects. No significant difference was observed between non-endogenomorphic depressed subjects and non-depressed subjects.

#### Multivariate analyses

Multiple regression analysis was performed on the PAS-CONS scale, using the PAS-CONS as dependent variable and the group (PD versus Controls) and age as independent variables or predictors.

A significant association was observed [*F*(2, 86) = 4.89, *p* < 0.01] for the two predictors. *t* values for group and age were *t*(86) = 2.54, *p* = 0.013 and *t*(86) = 2.31, *p* = 0.023), respectively.

### Relationship between anhedonia and depression in the OND group

Successive ANOVAs were performed using each anhedonia scale as dependent variable and each subtype of depression as independent variable. No significant association was observed between any of the anhedonia scales and gender, age, or level of education. None of these socio-demographic variables were therefore introduced into ANOVA as confounding variables.

Only one ANOVA was significant for TEPS-ANT [*F*(2, 37) = 3.81, *P* = 0.03]. *Post hoc* tests revealed that non-endogenomorphic depressed subjects had significant lower TEPS-ANT scores (i.e., were more severely anhedonic) than non-depressed subjects.

### Parkinson’s disease group

Univariate associations between each anhedonia scale and socio-demographic variables, depression and characteristics of the disease in the PD group are shown in Table 2. Twelve significant differences were observed, with a trend to significance for four values.

#### Univariate analyses

Analysis of socio-demographic variables showed that age was negatively associated with PAS-ANT, PAS-CONS, and SHAPS (*r* = −0.23, *p* < 0.1, −0.34, *p* < 0.05, −0.43, *p* < 0.05, respectively). Higher scores on these scales were observed in the youngest PD subjects. Higher PAS-ANT scores were observed in males compared to females (*p* < 0.09).

Analysis of clinical variables showed that age at onset was negatively associated with PAS-CONS, PAS-ANT, and SHAPS and duration of disease was negatively associated with anhedonia measured by TEPS-CONS (*r* = 0.45, *p* < 0.05) and SHAPS (*r* = −0.25 *p* < 0.05).

Higher stages of disease, as rated by Hoehn and Yahr stage, were associated with PAS-CONS (*r* = −0.32, *p* < 0.05) and TEPS-ANT (*r* = 0.28, *p* < 0.05). Motor impairment, as evaluated by the UPDR-III subscale, was associated with TEPS-ANT (*r* = 0.29, *p* < 0.05). MMSE score was associated with TEPS-CONS (*r* = 0.29, *p* < 0.05) and subjects who received entacapone had lower PAS-ANT scores than PD subjects not treated with this molecule.

Depression subtypes were associated with SHAPS scores. A significant difference was observed according to subtype of depression, classified as no depression, mild depression, and severe depression [*F*(2, 46) = 4.21, *p* < 0.021]. *Post hoc* tests showed that severely depressed subjects had significantly higher SHAPS scores than mildly depressed subjects, while the SHAPS scores of non-depressed subjects were not significantly different from those of mildly depressed subjects or severely depressed subjects. Analysis of subtypes of depression according to no depression, non-endogenomorphic depression, and endogenomorphic depression showed a trend toward significance [*F*(2, 46) = 2.46, *p* < 0.1]. *Post hoc* tests revealed a trend for endogenomorphic depressed subjects to have significantly higher SHAPS scores than non-endogenomorphic depressed subjects. The SHAPS scores of non-depressed subjects were not significant different from those of non-endogenomorphic depressed subjects or severe endogenomorphic depressed subjects.

#### Multivariate analyses

Duration of disease and MMSE scores were introduced into multiple regression analysis of the TEPS-CONS and no significant associations were observed. Hoehn and Yahr stage and UPDR-III scores were introduced into multiple regression analysis of the TEPS-ANT and no significant associations were observed. Age, age at onset, and Hoehn and Yahr stage were introduced into the regression analysis of the PAS-CONS. A significant association was observed [*F*(3, 45) = 10.6, *p* < 0.0003]. Among the three predictors, only age was significant [*t*(45) = 3.14, *p* < 0.0006] explaining 56% of the variance.

Four variables were entered into the regression analysis of the PAS-ANT: gender, age, age at onset, and entacapone. No significant association was observed.

Two multiple regression analyses were performed for the SHAPS. The first multiple regression analysis included age, age at onset, and depression subtype (no depression, mild depression, severe depression) as predictors and no significant association was observed. The second multiple regression analysis includes age, age at onset, and depression subtype (no depression, no endogenomorphic depression, endogenomorphic depression) as predictors and no significant association was observed.

## Discussion

This is the first study to examine the various components of anhedonia in Parkinsonian subjects, especially the distinction between anticipatory and consummatory anhedonia. This study was also characterized by three important points not taken into account in previous studies. Firstly, the control group was composed of patients with motor non-PD neurologic disease. The use of this type of control group allowed control of motor impairment as a potential bias that could lead to anhedonia secondary to reactive depression. Secondly, socio-demographic characteristics (age, gender, level of education, inpatient/outpatient status) were statistically controlled. Thirdly, the distinction between endogenomorphic and non-endogenomorphic depression was taken into account.

The control group of the present study was composed of patients presenting dystonia, cerebellar, or peripheral nervous system diseases. Generally, dystonia is considered as basal ganglia disorder and abnormalities in dopaminergic activity have been proposed. The inclusion of such patients in the control group might have influenced the results. This potential bias has been discarded as the patients with dystonia did not differ on the socio-demographic, clinical, and psychometrical variables comparatively with the other controls.

It is important to note that the OND group allowed the control of motor impairment and its consequences as reactive depression. Among the eight previous published studies (5–12), any study has used non-PD neurological patients as controls.

### Our first hypothesis tested whether one particular type of anhedonia specifically characterized PD subjects

Univariate and multivariate analyses on the two samples showed that physical consummatory anhedonia was characteristic of PD subjects. This effect is independent of age and depression, as demonstrated by multivariate analyses. This result was unexpected, as we had initially hypothesized that higher levels of anticipatory anhedonia would be observed in PD subjects compared to subjects with non-PD motor neurologic disease. Consummatory anhedonia, as measured by decreased sucrose preference in rats, requires combined depletion of all three monoamines (serotonin, dopamine, noradrenaline) (26). Physical consummatory anhedonia in PD could therefore be related and explained by dopamine, noradrenaline, and serotonin deficiency. If these high levels of consummatory anhedonia are confirmed by other studies, agonists of these three neurotransmitters could therefore be used to treat anhedonia in PD subjects. Another explanation could be related with the use of a control group including subjects with dystonia. In this disorder, dopaminergic deficiency has been involved suggesting that the level of anticipatory anhedonia could be the same between the PD and control groups.

The prevalence of anhedonia using the SHAPS cutoff in this study did not differ between the two groups and was 18.4% in the PD group. This value was similar to those reported in previous studies, ranging from 4.7% (12) to 45.7% (7).

The second hypothesis tested the relationship between anhedonia and depression and especially the association between consummatory or anticipatory anhedonia and non-endogenomorphic or endogenomorphic forms of depression.

Firstly, only the SHAPS was significantly associated with this depression subtype. This association was observed for the entire sample and in PD subjects. Endogenomorphic depressed subjects had higher SHAPS scores than non-endogenomorphic depressed subjects. As the SHAPS score rates consummatory and anticipatory pleasure, the present study did not confirm our *a priori* hypothesis of a relationship between anticipatory anhedonia and non-endogenomorphic depression and between consummatory anhedonia and endogenomorphic depression. However, our results were compatible with the hypothesis formulated by Klein (13). This author suggested that the defect in the consummatory pleasure system was always accompanied by a defect in the appetitive (anticipatory) pleasure system. Secondly, Klein suggested that a defect in the consummatory system always produced autonomous or endogenomorphic depression. The relationships between consummatory and anticipatory anhedonia and non-endogenomorphic versus endogenomorphic depression are therefore not specific to PD (27).

Moreover, for the entire sample and for PD subjects, only the SHAPS was significantly associated with depression subtype, classified as no depression, mild depression, and severe depression. Severely depressed subjects had significantly higher SHAPS scores than mildly depressed subjects. The results of this study therefore show that the relationship between depression and anhedonia is not specific to PD.

Secondly, only severely depressed or endogenomorphic depressed subjects had high levels of anhedonia compared to non-endogenomorphic depressed or mildly depressed subjects. These results suggest that mildly depressed subjects with motor (PD or other) neurologic diseases were not characterized by high levels of anhedonia. Moreover, these results were observed for consummatory and anticipatory anhedonia rated together. Several studies using the SHAPS or PAS scales have reported either no relationship between anhedonia and depression (6) or higher levels of anhedonia in depressed PD subjects than in controls (7, 9). However, in these studies, the authors did not distinguish between non-depressed, mildly depressed, and severely depressed subjects. A recent study conducted in 254 PD subjects compared SHAPS scores between non-depressed PD subjects, mildly depressed subjects, and severely depressed subjects (12), in whom depression was diagnosed according to DSM-IV criteria. Severely depressed PD subjects had higher SHAPS scores than the other two groups. These authors also reported significant correlations between the SHAPS and BDI-II scores only in the severely depressed and non-depressed PD groups and concluded that anhedonia, as rated by the SHAPS, was correlated with the severity of depression in PD subjects with either severe depression or no depression.

We therefore propose that high levels of consummatory and anticipatory anhedonia, as rated by the SHAPS, could characterize endogenomorphic or severely depressed PD subjects compared to non-endogenomorphic or mildly depressed PD subjects. However, this relationship was also observed in patients with non-PD motor neurologic disease. We did not confirm the results of the study by Spalletta et al. (12) that suggested that non-depressed PD subjects were characterized by anhedonia.

Relationships between SHAPS and depression have been explored in healthy or psychiatric subjects. Several studies, using the BDI, reported significant correlations with higher values in psychiatric subjects than in healthy subjects (28, 29).

The third hypothesis examined the relationships between features of the disease in the PD group and the various types of anhedonia in order to detect a specific pattern of associations, especially explaining the discrepancies reported in the literature. Univariate analyses revealed several significant or non-significant associations between anhedonia scales and clinical variables, but none of the multivariate analyses performed for each anhedonia scale were significant. The present study therefore failed to demonstrate a particular profile between each anhedonia scale and clinical variables.

Two studies using the SHAPS and one study using the TEPS-ANT and TEPS-CONS did not observe any relationship between anhedonia and age at onset or duration of disease (10, 11). One study did not report any relationship between age at onset, duration of disease, Hoehn and Yahr stages, and SHAPS scores (11) and Isella et al. (6), using the PAS scale, did not observe any significant correlation between the scale and age, UPDR-III, or disease duration. Moreover, no relationship has been reported between UPDRS-III, Hoehn and Yahr stage, or disease duration and TEPS-ANT and TEPS-CONS (11).

No relationship has been reported between MMSE scores and SHAPS (10) or TEPS-CONS or TEPS-ANT (11). One study found an association between entacapone and more severe anhedonia, as rated by the SHAPS (10).

It is noteworthy that all but one (10) of the previous studies reporting significant associations between anhedonia scales and clinical characteristics of the disease did not control for socio-demographic variables and the other significant clinical variables by means of multivariate analyses such as multiple regression analysis.

In the present study, multivariate analyses did not provide any significant results except for the PAS-CONS. Age was significantly associated with consummatory physical anhedonia (30). Young PD subjects were characterized by high levels of this type of anhedonia.

This study has a number of limitations. Firstly, the small sample sizes may explain the negative results due to the low power of statistical tests. Secondly, PD subjects were consecutive outpatients presenting various stages of PD. PD subjects represented a heterogeneous sample that may have presented various levels of hedonic deficits.

## Conclusion

Anhedonia in PD could be explained by socio-demographic variables, depression, and characteristics of the disease. There are not currently accepted treatment strategies for the different anhedonias. The present study suggests, for the first time, that physical consummatory anhedonia could be specific to PD in contrast with anticipatory anhedonia.

## Conflict of Interest Statement

The authors declare that the research was conducted in the absence of any commercial or financial relationships that could be construed as a potential conflict of interest.

## References

[1] Aarsland D Marsh L Schrag A Neuropsychiatric symptoms in Parkinson’s disease. Mov Disord (2009) 24:2175–86 10.1002/mds.22589 19768724 PMC2787875

[2] Winograd-Gurvich C Fitzgerald PB Georgiou-Karistianis N Bradshaw JL White OB Negative symptoms: a review of schizophrenia, melancholic depression and Parkinson’s disease. Brain Res Bull (2006) 70:312–21 10.1016/j.brainresbull.2006.06.007 17027767

[3] Chaudhuri KR Schapira AHV Non-motor symptoms of Parkinson’s disease: dopaminergic pathophysiology and treatment. Lancet Neurol (2009) 8:464–74 10.1016/S1474-4422(09)70068-7 19375664

[4] Loas G Salinas E Guelfi JD Samuel-Lajeunesse B Physical anhedonia in major depressive disorder. J Affect Disord (1992) 25(2):139–46 10.1016/0165-0327(92)90076-I 1644989

[5] Pluck GC Brown RG Apathy in Parkinson’s disease. J Neurol Neurosurg Psychiatry (2002) 73:636–42 10.1136/jnnp.73.6.636 12438462 PMC1757348

[6] Isella V Iurlaro S Piolti R Ferrarese C Frattola L Appollonio I Physical anhedonia in Parkinson’s disease. J Neurol Neurosurg Psychiatry (2003) 74:1308–11 10.1136/jnnp.74.9.1308 12933942 PMC1738679

[7] Lemke MR Brecht HM Koester J Kraus PH Reichmann H Anhedonia, depression, and motor functioning in Parkinson’s disease during treatment with pramipexole. J Neuropsychiatry Clin Neurosci (2005) 17:214–20 10.1176/appi.neuropsych.17.2.214 15939976

[8] Santangelo G Morgante L Savica R Marconi R Grasso L Antonini D Anhedonia and cognitive impairment in Parkinson’s disease: Italian validation of the Snaith-Hamilton Pleasure Scale and its application in the clinical routine practice during the PRIAMO study. Parkinsonism Relat Disord (2009) 15:576–81 10.1016/j.parkreldis.2009.02.004 19362509

[9] Fujiwara S Kimura F Hosokawa T Ishida S Sugino M Hanafusa T Anhedonia in Japanese patients with Parkinson’s disease. Geriatr Gerontol Int (2011) 1:275–81 10.1111/j.1447-0594.2010.00678.x 21241445

[10] Miura S Kida H Nakajima J Noda K Nagasato K Ayabe M Anhedonia in Japanese patients with Parkinson’s disease: analysis using the Snaith-Hamilton Pleasure Scale. Clin Neurol Neurosurg (2012) 114:352–5 10.1016/j.clineuro.2011.11.008 22137783

[11] Zahodne LB Marsiske M Okun MS Bowers D Components of depression in Parkinson disease. J Geriatr Psychiatry Neurol (2012) 25(3):131–7 10.1177/0891988712455236 22859701 PMC3490054

[12] Spalletta G Fagioli S Meco G Pierantozzi M Stefani A Pisani V Hedonic tone and its mood and cognitive correlates in Parkinson’s disease. Depress Anxiety (2013) 30(1):85–91 10.1002/da.22036 23300113

[13] Klein DF Depression and anhedonia. In: Clark DC Fawcett J editors. Anhedonia and Affect Deficit States. New York: PMA Publishing Corp (1987). p. 1–14

[14] Gard DE Germans Gard M Kring AM John OP Anticipatory and consummatory components of the experience of pleasure: a scale development study. J Res Pers (2006) 40:1086–102 10.1016/j.jrp.2005.11.001

[15] Burgdorf J Panksepp J The neurobiology of positive emotions. Neurosci Biobehav Rev (2006) 30:173–87 10.1016/j.neubiorev.2005.06.001 16099508

[16] Gard DE Kring AM Germans Gard M Horan WP Green MF Anhedonia in schizophrenia: distinction between anticipatory and consummatory pleasure. Schizophr Res (2007) 93:253–60 10.1016/j.schres.2007.03.008 17490858 PMC1986826

[17] Loas G Monestes JL Ameller A Bubrovszky M Yon V Wallier J Traduction et étude de validation de la version française de l’échelle d’expérience temporelle du plaisir [EETP, Temporal Experience of Pleasure Scale (TEPS), Gard et al., 2006]: étude chez 125 étudiants et chez 162 sujets présentant un trouble psychiatrique. Ann Med Psychol (2009) 167:641–8

[18] Chapman LJ Chapman JP Raulin M Scales for physical and social anhedonia. J Abnorm Psychol (1976) 85:374–82 10.1037/0021-843X.85.4.374 956504

[19] Loas G Boyer P Evaluation of anhedonia in psychopathology: second study of the validation of the French version of the Chapman and Chapman physical anhedonia scale. Study of 356 persons. Ann Med Psychol (Paris) (1994) 152:256–9 8092667

[20] Snaith RP Hamilton M Morley S Humayan A A scale for the assessment of the hedonic tone: the Snaith-Hamilton pleasure scale. Br J Psychiatry (1995) 167:99–103 10.1192/bjp.167.1.99 7551619

[21] Leentjens AFG Dujardin K Marsh L Martinez-Martin P Richard IH Starkstein SE Apathy and anhedonia rating scales in Parkinson’s disease: critique and recommendations. Mov Disord (2008) 23:2004–14 10.1002/mds.22229 18709683

[22] Loas G Dubal S Perot P Tirel F Nowaczkowski P Pierson A Validation of the French version of the Snaith-Hamilton Pleasure Scale (SHAPS, Snaith et al. 1995). Determination of the statistical parameters in 208 normal subjects and 103 hospitalized patients presenting with depression or schizophrenia. Encephale (1997) 23:454–8 9488929

[23] Beck AT Steer RA Ball R Ranieri W Comparison of beck depression inventories-IA and -II in psychiatric outpatients. J Pers Assess (1996) 67:588–97 10.1207/s15327752jpa6703_13 8991972

[24] Centre de Psychologie Appliquée X Validation de la Version Française du BDI-II. Paris: Editions du Centre de Psychologie Appliquée (1996).

[25] Haslam N Beck AT Subtyping major depression: a taxometric analysis. J Abnorm Psychol (1994) 103:686–92 10.1037/0021-843X.103.4.686 7822569

[26] Delaville C Chetrit J Abdallah K Morin S Cardoit L De Deurwaerdère P Emerging dysfunctions consequent to combined monoaminergic depletions in Parkinsonism. Neurobiol Dis (2012) 45:763–73 10.1016/j.nbd.2011.10.023 22079236

[27] Loas G Boyer P Anhedonia in endogenomorphic depression. Psychiatry Res (1996) 60:57–65 10.1016/0165-1781(95)02748-3

[28] Nakonezny PA Carmody TJ Morris DW Kurian BT Trivedi MH Psychometric evaluation of the Snaith-Hamilton pleasure scale in adult outpatients with major depressive disorder. Int Clin Psychopharmacol (2010) 25(6):328–33 10.1097/YIC.0b013e32833eb5ee 20805756 PMC2957191

[29] Liu WH Wang LZ Zhu YH Li MH Chan RCK Clinical utility of the Snaith Hamilton Pleasure scale in Chinese settings. BMC Psychiatry (2012) 12:184 10.1186/1471-244X-12-184 23110667 PMC3549923

[30] Loas G Adaptation et validation française de l’échelle d’anhédonie physique de Chapman et Chapman: [physical Anhedonia Scale (PAS)] Adaptation and French validation of the Physical Anhedonia Scale: PAS (Chapman and Chapman, 1978). Encephale (1993) 19(6):639–44 12404783

